# Citizen science: A new perspective to advance spatial pattern evaluation in hydrology

**DOI:** 10.1371/journal.pone.0178165

**Published:** 2017-05-30

**Authors:** Julian Koch, Simon Stisen

**Affiliations:** 1 Department of Hydrology, Geological Survey of Denmark and Greenland, Copenhagen, Denmark; 2 Department of Geosciences and Natural Resources Management, University of Copenhagen, Copenhagen, Denmark; Bristol University/Remote Sensing Solutions Inc., UNITED STATES

## Abstract

Citizen science opens new pathways that can complement traditional scientific practice. Intuition and reasoning often make humans more effective than computer algorithms in various realms of problem solving. In particular, a simple visual comparison of spatial patterns is a task where humans are often considered to be more reliable than computer algorithms. However, in practice, science still largely depends on computer based solutions, which inevitably gives benefits such as speed and the possibility to automatize processes. However, the human vision can be harnessed to evaluate the reliability of algorithms which are tailored to quantify similarity in spatial patterns. We established a citizen science project to employ the human perception to rate similarity and dissimilarity between simulated spatial patterns of several scenarios of a hydrological catchment model. In total, the turnout counts more than 2500 volunteers that provided over 43000 classifications of 1095 individual subjects. We investigate the capability of a set of advanced statistical performance metrics to mimic the human perception to distinguish between similarity and dissimilarity. Results suggest that more complex metrics are not necessarily better at emulating the human perception, but clearly provide auxiliary information that is valuable for model diagnostics. The metrics clearly differ in their ability to unambiguously distinguish between similar and dissimilar patterns which is regarded a key feature of a reliable metric. The obtained dataset can provide an insightful benchmark to the community to test novel spatial metrics.

## 1. Introduction

There is a growing number of scientific projects that actively include members of the general public in their research. In such projects, a large number of non-specialists perform a wide range of mostly relatively simple task such as image analysis, pattern recognition, document transcription or data collection [1]. This type of active involvement of the general public is referred to as citizen science and currently it receives rising attention from the scientific community, policy makers and funding agencies [2]. Franzoni and Sauermann [2] provide an excellent overview of the organizational structures and key features of citizen science. In brief summary, citizen science can be discriminated from traditional science by openness in participation and disclosure of intermediate results.

There exist resourceful internet based infrastructure that allows researchers to build citizen science projects and to reach large numbers of active users. A noteworthy example is Zooniverse; a platform that provides an excellent infrastructure to build, host and manage citizen science projects [3]. Most of Zooniverse’s projects originate from the broad spectrum of natural sciences and outsource visual tasks of all kinds to its users [4–6]. It facilitates an interactive design where users can easily contribute to science and communicate with each other and the researchers. Furthermore, the researchers can engage with the community by disclosing intermediate results.

Up to present, the field of hydrology understands citizen science exclusively as an alternative way to collect hydrological data by establishing so called citizen observatories [7]. Buytaert et al. [8] provided a broad overview of successfully implemented citizen observatories that collect data on hydrological variables such as precipitation, streamflow, water quality and others. The advent of cheap, robust and low-maintenance sensing techniques enables this outsourcing that effectively complements traditional ways of monitoring. The trustworthiness and quality of data generated by non-specialists is an ongoing discussion among the citizen science community [9–11].

Despite these previous advances in the field of hydrology, the full potential of human capacities is generally overlooked. Cooper at al. [12], Khatib et al. [13] and others argued that humans, supported by their intuition and reasoning, can outperform computers in several realms of problem solving. Humans are also considered to have a superior capacity to evaluate spatial data, because we automatically recognize and interpret multiple features of spatial information at different scales. As an example, Cloke and Pappenberger [14] relied on “eye balling” to evaluate computer algorithms that provide a performance measure of meteorological predictions. Furthermore, Wealands et al. [15] suggested to emulate the human visual perception when applying quantitative measures to compare spatial patterns of soil moisture. Recently, Koch et al. [16] benchmarked a set of spatial performance metrics against the human perception which was quantified by means of a web-based survey where participants rated spatial similarity between synthetic patterns of land-surface-temperature. The above mentioned studies shared two key conclusions: (1) There is a clear need to quantitatively compare spatial patterns in order to better integrate spatial information from e.g. remote sensing observations in distributed hydrological models. (2) The human perception can complement such metrics but eventually cannot serve in automatized procedures such as model calibration, because the large number of model evaluations can only be conducted by predefined metrics. Furthermore human subjectivity can potentially bias the evaluation of spatial patterns. Nevertheless, metrics that mimic the human perception should be favored in order to grant a meaningful pattern evaluation.

Citizen science can bundle the visual comparison skills of thousands of volunteers and can serve as a benchmark to evaluate metrics with respect to their ability to distinguish between similar and dissimilar spatial patterns. This study highlights a citizen science project that employs the well trained human visual perception to compare spatial patterns of hydrological variables. The spatial patterns originate from a hydrological model of a mesoscale catchment (~2500 km^2^) that is additionally coupled to a land-surface model to better represent the complex spatio-temporal variability at the land-atmosphere interface. Spatial patterns of three core land-surface variables, namely daily actual evapotranspiration (ET), daily land-surface temperature (LST) and land-surface temperature rise between nighttime and daytime (LSTr) are featured in the project with the aim to identify the predominant drivers behind these simulated spatial patterns.

Many environmental problems are spatially explicit and robust distributed models are required to tackle the associated challenges. Along these lines, the relevancy of spatial patterns in hydrology has been intensively reported in literature [17–19]. Aggregated variables, such as stream discharge at the outlet of a catchment, are not an appropriate measures of spatial variability of the hydrological processes within the catchment [20–22]. This highlights the need to include spatial observations. In recent years, several studies proposed spatial performance metrics that enable a meaningful pattern comparison of hydrological variables that go beyond simple cell-to-cell comparisons [15,23,24]. This metric requirement is favorable because of associated uncertainties at the individual grid cell in both model and observation as well as differences in scale between them. Further, in the light of spatial model calibration, spatial metrics are attentive to generate more realistic spatial distributions of parameter fields. For the purpose of this study, we have assembled a set of advanced metrics that includes (1) a connectivity analysis [25], (2) an empirical orthogonal functions analysis [16], (3) a fraction skill scores assessment [26] and (4) a variogram analysis [27]. In addition, two simple but well known cell-to-cell metrics, i.e. root-mean-squared-error and Pearson's correlation coefficient, are included to investigate if more complex metrics that are specifically tailored to evaluate spatial patterns outperform simpler metrics.

In summary, the main objective of this study is to utilize citizen science to learn from the human perception on how to better evaluate spatial patterns in hydrological modelling. Implications are investigated twofold; (1) to evaluate a set of advanced spatial performance metrics against the human perception in their ability to mimic the human vision and (2) to use citizen science in combination with the set of metrics in a spatial sensitivity analysis to identify the predominant drivers of spatial variability at the land-atmosphere interface of a hydrological model.

## 2. Methods & data

### 2.1. Study site

**Fig 1:**
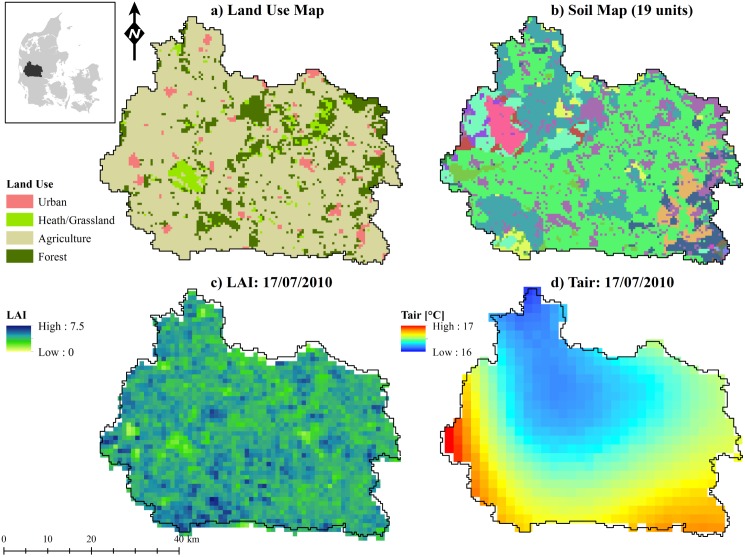
The Skjern river catchment in western Denmark. a) Land use map with the four predominant land use classes, b) soil map with 19 soil units (legend not shown), c) MODIS derived Leaf Area Index (LAI) snapshot in July 2010 and d) average air temperature (Tair) on the same day based on interpolated climate station data from inside and outside of the catchment.

The Skjern river catchment is located in the western part of the Danish peninsula and its size amounts to 2500 km^2^. The catchment has been studied intensively for almost a decade by the HOBE project [28]. The climate is maritime with a mean annual precipitation of 990 mm of which 575 mm constitute the mean annual reference evapotranspiration. Soils are predominately sandy with intertwined till and clay sections. Topography slopes gently from the highest point of approximately 125 m elevation in the east to sea level in the western side of the catchment. Agriculture is the predominant land use in the Skjern catchment followed by forest, heath and urban areas, as indicated by the land use map in Fig 1A).

### 2.2. Hydrological model

#### 2.2.1 Model setup

The MIKE SHE modelling system is utilized to set up a catchment model of the Skjern river catchment. MIKE SHE is a distributed process based hydrological model that comprises fully coupled modules of 3D saturated flow (finite difference), 1D unsaturated flow (Richards’ equation), river routing and overland flow [29]. In order to further enhance process detail, a land-surface model that solves the diurnal energy balance (SW-ET [30]) is coupled to the traditional modelling system. This grants a better representation of temporal variability and spatial patterns of the relevant processes at the land-atmosphere interface. Land-surface modelling with SW-ET requires hourly climate forcing (air temperature, humidity, wind speed, radiation and air pressure) and time steps to reflect the complex diurnal variability correctly [31]. In addition, a detailed vegetation parametrization scheme is required. Information on leaf area index (LAI), root depth, albedo and vegetation height are derived from the Moderate Resolution Imaging Spectroradiometer (MODIS) and details are presented by Koch et al. [32]. Other vegetation parameters such as interception coefficient or stomata resistance are given for each of the main land use classes as shown in Fig 1A) and plausible values are derived from literature [33]. Daily precipitation fields are generated on the basis of 43 rain gauges within and around the catchment. The Skjern model is set up for the period of 2002 to 2010 at 500 m resolution and this study investigates the simulated spatial patterns of the last three years. For a more detailed description of the model setup and model calibration we refer to Stisen et al. [22] and Koch et al. [32]

#### 2.2.2. Model scenarios

The Skjern catchment model contains pronounced spatial detail in all required model parameters and forcing data. Fig 1 illustrates this by showing a) the land use map that is partly utilized for the vegetation parametrization, b) the soil map that comprises 19 units; each with individual van Genuchten parameters, c) a snapshot of MODIS derived LAI which is available every 8 days at 1 km spatial resolution and d) a daily average air temperature that is interpolated to 2 km based on climate station data.

A baseline model and six scenarios build the basis for the spatial pattern evaluation in this study. The baseline model fully integrates all available spatial detail in parameters and forcing data. Each of the six scenarios reflects a deterioration of a potential driver of spatial variability and Table 1 lists the perturbation strategies. Simulated spatial patterns from the scenarios are evaluated against the baseline model on the premise that if a perturbation in spatial input alters the simulated spatial patterns significantly, the model is deemed sensitive to that specific driver. Vice versa, little change in the simulated patterns of a scenario indicates limited sensitivity [32]. Three land-surface variables are selected for the sensitivity analysis, namely daily actual evapotranspiration (ET), daily land-surface-temperature (LST) and land-surface-temperature rise (LSTr) which quantifies the heating rate between nighttime and daytime LST. Table 1 groups the six scenarios into climate-, soil/geology- and vegetation-relevant. In scenarios 1, 2, 4 and 6, the respective parameters or forcing data are spatially averaged to constant values for the entire catchment. As an intermediate step, scenario 5 constitutes a moving average 10 km smoothing filter applied to the MODIS derived vegetation parameters. Lastly, scenario 3 addresses the groundwater influence of spatial patterns at the land-surface. In this scenario, the conceptual model setup is simplified by disabling the saturated zone and setting a constant groundwater head boundary below the root depth. A better understanding of the most critical spatial processes at the land-atmosphere interface will guide the modelling community to properly diagnose spatial model deficiencies and to efficiently incorporate spatial information (e.g. by means of remote sensing) to model parameters and forcing.

**Table 1:** Overview of the six model scenarios investigated in this study.

Module	Scenario#	Forcing Data / Parameter	Original Resolution	Spatial Perturbation	Temporal
**Climate**	1	Precipitation	500 m	set constant	Yes
	2	Air temperature, humidity, pressure, wind speed and radiation	2 km		
**Soil + Geology**	3	Disable groundwater coupling	500 m	fixed head below RD	No
	4	Soil map and top 3m geological layer		Set to predominant class	
**Vegetation**	5	Leaf area index, root depth, albedo, and vegetation height	1 km	10 km smoothing	Yes
	6			set constant	

### 2.3. Citizen science: Human visual perception

**Fig 2:**
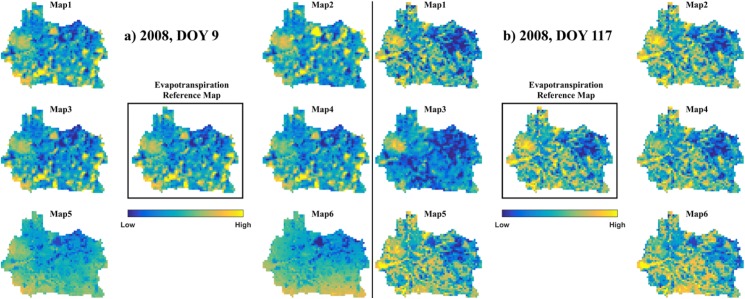
Two subjects, taken from the citizen science project: Simulated daily evapotranspiration maps at two days in 2008. The center map originates from the baseline model and map 1 to 6 originate from scenario 1 to 6, respectively.

In order to effectively learn from the human perception we have built a Zooniverse project, entitled *Pattern Perception*, which was officially released on May 23^rd^ 2016. Prior to the release it has been reviewed by the Zooniverse team, who ensured that it complied with their rules and requirements (https://www.zooniverse.org/lab-policies). Additionally, Zooniverse users that have consented to their availability for beta-testing reviewed the project’s quality. *Pattern Perception* asks the Zooniverse users to classify spatial similarity and dissimilarity between the six scenarios and the baseline model that are introduced in section 2.2.2. The users help to identify sensitive scenarios that significantly alter the simulated spatial patterns at the land-surface of the Skjern model and vice versa, scenarios that are insensitive and hence do not affect the spatial patterns of ET, LST and LSTr. More importantly the collective similarity scores can subsequently be utilized to test and benchmark spatial performance metrics. Spatial patterns for each of the three variables are investigated every third day between 2008 and 2010 which results in 365*3 subjects. A subject can be understood as an image which constitutes spatial patterns of the six scenarios and the baseline model of one of the three variables at a specific day. Fig 2 illustrates the comparison between ET maps simulated by the six scenarios and the baseline model for two selected days, which resembles the layout of the images used in the actual project. The project contains two tasks which are presented independently from each other: The users are asked to classify either (1) the most similar or (2) the most dissimilar scenario with respect to the baseline model which is shown as the reference in the centre of the image. Classification is conducted by simply clicking on the maps, which are then marked by a crosshair. If it is not possible for the users to identify a single map that is most similar or dissimilar they are encouraged to classify multiple which they regarded as equally most similar or dissimilar. Consequently, each subject needs two classifications, similarity and dissimilarity, which results in 365*3*2 subjects. We have decided to clearly separate these two tasks from each other in order to eliminate the chance of potential misclassifications as much as possible. An anticipated source of misclassifications is for example users that loose track due to a constant change of classifying similarity and dissimilarity. The retirement limit, which defines a self-proclaimed goal of classifications for each subject, is set to 20, resulting in 43800 (365*3*2*20) required classifications in total. The users are presented with images containing patterns of the six scenarios and the baseline model for each day; as shown in Fig 2. Unlike Fig 2 where the six scenarios are in order with respect to Table 1, the order of the scenarios is randomly shuffled in the final subjects that are uploaded to Zooniverse.

A simple distance matrix is utilized to interpret the classifications which reflect the collective visual perception of the users. The distance (*d*) reflects the degree of spatial similarity of each scenario to the baseline relative to the other scenarios. Starting with *d* = 0, the similarity and the dissimilarity classifications are merged for each subject. Each scenario receives *d*+1 for each similarity classification and *d*-1 for each dissimilarity classification. Considering all classifications, the most similar scenario is consequently flagged with the highest *d* and the most dissimilar scenario is marked with the lowest *d*. As a last step, *d* is normalized to range between 0 (most dissimilar scenario) and 1 (most similar scenario), where the former presents the most sensitive scenario and the latter the most insensitive scenario. The normalization is achieved by dividing the distance matrix of a particular subject by its highest *d*. This results in a quantitative pattern similarity score based on a collective assessment of the human perception. Caution has to be exercised when comparing the scores of different subjects, because it essentially reflects a relative ranking of the six scenarios with respect to the baseline for a specific subject.

### 2.4. Spatial performance metrics

This study features four spatial performance metrics that provide a meaningful evaluation of simulated spatial patterns against a reference. An exhaustive description of the applied metrics is given by Koch et al. [32] and, in order to avoid repetition, we only provide a brief introduction to each metric. In addition, two well-known and straightforward metrics namely, root-mean-square-error (RMSE) and Pearson’s correlation coefficient (spatial R) are included and compared to the more complex metrics introduced below.

#### 2.4.1. Empirical orthogonal functions

The empirical orthogonal functions (EOF) analysis is a popular methodology to decompose the spatio-temporal variability of hydrological variables such as soil moisture [34], groundwater table [35] or LST [36]. Following a detailed technical description by Perry and Niemann [37], the EOF analysis decomposes a large spatio-temporal dataset into two components: 1) a set of orthogonal EOF maps that are invariant in time and 2) a set of loadings that quantify the oscillation of each EOF over time. The linear combination of the first EOF map and the corresponding loadings always explains the highest portion of variance. Recently, the EOF analysis has gained attention as a tool to spatially validate distributed hydrological models at catchment scale [38] and continental scale [23]. In particular, Koch et al. [16] highlighted the benefits of a joint analysis on both, reference and simulated data which guarantees that the obtained EOF maps honour the spatio-temporal variability of both datasets. Hence, the deviation between the loadings at a certain timestep can be utilized as an indicator of spatial similarity. Following this approach, the associated loadings of highly similar maps at a certain timestep will show a minimal deviation. Vice versa, a large loading deviation can be attested to pattern dissimilarity.

#### 2.4.2. Fractions skill score

The fractions skill score (FSS) is commonly applied by meteorologists to obtain a scale dependent metric that quantifies spatial skill of various competing precipitation forecasts with respect to a reference [24,26,39]. A fraction reflects the occurrence of values exceeding a certain threshold at a given window size *n* and is calculated at each cell. Typically the thresholds are derived from the variable’s percentiles, which constitutes the bias insensitivity of FSS [40]. The FSS methodology is defined by three steps: (1) for each threshold, truncate the spatial patterns of the baseline and a scenario into binary maps, (2) for each cell, compute the fraction of cells that exceed the threshold and lie within a window of size n*n and (3) calculate the mean-squared-error (MSE) between the reference and scenario fractions and normalize it with a worst case MSE that reflects the condition with zero agreement between the spatial patterns. FSS ranges from zero to one, where one indicates a perfect agreement between baseline and scenario patterns and zero reflects the worst possible performance. For the simulated spatial patterns in the Skjern catchment we selected three top and three bottom percentiles and each is assessed at an individual critical scale. The 1^st^, 5^th^ and 20^th^ percentiles focus on the bottom 1%, 5% and 20% of cells and are investigated at 25 km, 15 km and 5 km scale, respectively. Three top percentiles, 99^th^, 95^th^ and 80^th^ are analysed analogous. The average of the three top and bottom percentiles is calculated as an overall pattern similarity score and referred to as *FSS top* and *FSS bottom* in the following analysis.

#### 2.4.3. Connectivity

The connectivity metric originates from the field of hydrogeology where it is commonly applied to characterise aquifer heterogeneity [41–43]. Only a few studies have brought the concept to the land-surface community and applied it on soil moisture patterns [44,45] and LST patterns [23]. Following Renard and Allard [25], the connectivity analysis of a continuous variable is conducted via three steps: (1) a series of threshold percentiles decomposes the domain into a series of binary maps, (2) the binary maps undergo a cluster analysis that identifies connected clusters and (3) the transition from many disconnected clusters to a single connected clusters can be quantified by principles of percolation theory [46]. A suitable percolation metric is the probability of connection that states the proportion of pairs of cells that are connected among all possible pairs of connected cells of a cluster map. The percolation is best described by means of an increasing threshold that moves along all percentiles of the variable’s range. This assures bias insensitivity and it allows investigating the clusters of high and low values separately. The connectivity analysis is applied individually on cells that exceed a given threshold and those that fall below. In the following, the two approaches are referred to as high and low phase, respectively. The root-mean–square-error between the connectivity at all percentiles of the baseline pattern and a scenario denotes a tangible pattern similarity metric and can be calculated individually for the high and the low phase and is referred to as *Con high* and *Con low*, respectively.

#### 2.4.4. Variogram

The variogram analysis is a frequently applied geostatistical tool to quantify the spatial autocorrelation structures of spatial data as a function of distance [47]. In land-surface hydrology, variograms are most commonly applied on soil moisture data to analyse observed spatial patterns [48,49] or to evaluate catchment models against observed patterns [19,20]. In previous studies, variograms of other core land-surface variables, such as ET, LST or LSTr are typically not considered. The omnidirectional empirical semivariance (γ) describes the autocorrelation of a variable at specific lags, which define the distance between cells. For the analysis of spatial patterns in the Skjern catchment, γ is calculated at eight unregularly spaced lags from 1 km up to 25 km, which is about half of the maximum possible lag distance in the catchment. In order to quantify the spatial similarity of the baseline and a scenario at a certain day, the root-mean–square-error between the γ at the eight given lags is computed to serve as a metric. In summary, the variogram metric is a global measure that is not constrained by local agreement. Furthermore the metric is bias insensitive, because it only considers the relative deviation between values.

## 3. Results & discussion

### 3.1. User statistics

The citizen science project *Pattern Perception* was officially launched on the Zooniverse platform on May 23^rd^, 2016. The self-proclaimed goal of 43,800 classifications was reached after64 days and Table 2 summarizes the classification statistics. Classifying similarity has been the more popular choice among the users which was most likely caused by the trivial reason that the layout of the project featured classifying similarity as the first option to choose from.

**Table 2:** User statistics for the citizen science project *Pattern Perception*. Data was obtained within 64 days after launch.

		# of maps	# of classifications	mean # of classifications
**ET**	similarity	365	8234	22.56
	dissimilarity	365	7406	20.29
**LST**	similarity	365	8057	22.07
	dissimilarity	365	7482	20.50
**LSTr**	similarity	365	8002	21.92
	dissimilarity	365	7504	20.56

**Fig 3:**
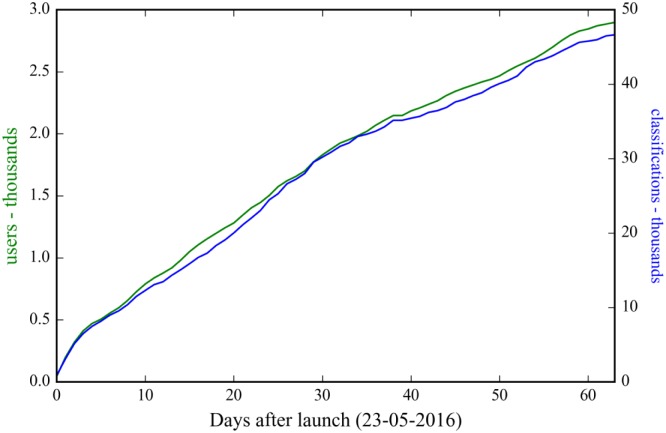
The cumulative user statistics for the citizen science project: The number of users (in green) and the number of classifications (in blue), both given in thousands.

The cumulative curves of user count and classification count are given in Fig 3 and reveal a rapid increase during the first week after launch followed by a more or less constant growth of approximately 40 new users a day and around 650 additional classifications a day. The user count has to be interpreted with care, because only registered users are counted uniquely. Opposed, returning unregistered users, that mark approximately half of the users, are counted multiple times according to their IP-address. Hence the real user count can be expected to be somewhat smaller. After 64 days the final turnout is 2,898 users that supplied 46,636 classifications in total.

Sauermann and Franzoni [50] analysed the user statistics of seven Zooniverse projects and revealed that typically only a small number of users are responsible for a large share of the classifications. This skewed distribution was quantified by the amount of classifications that was supplied by the top 10% of most active users and was found to vary between 71% and 88%. In the case of *Pattern Perception*, the top 10% of users contributed 65% of the total amount of classifications. The fact that the distribution is less skewed than the other Zooniverse projects may be related to the fact that the task in *Pattern Perception* is considered easy and classifications can be done very swiftly. Nevertheless there are around 16% of users that contributed with only a single classification of which 72% were unregistered users.

### 3.2. Survey interpretation

The proposed method on how to collectively merge the similarity and dissimilarity classifications into a relative spatial similarity score is described in section 2.3 and detailed results are stated in Table 3 for two example subjects (Fig 2). The first example (Fig 2A)) depicts a typical wintertime ET pattern of the Skjern catchment under energy limited conditions. As underlined by Table 3, most of the 20 users that addressed pattern similarity identified maps 1, 3 and 4 as being most similar. This stresses that many users found the given maps equally similar and thus supplied multiple classifications. Map 2 is more disputed as it is marked most similar by only approximately half of the users. In total 21 users rated map 5 and 6 as most similar with 19 classifications each. The ET pattern in the second example (Fig 2B)) represents a day in spring at the beginning of the growing season in the Skjern catchment. The pattern is generally more complex and the classifications are partly conflicting. The most similar and most dissimilar maps are clearly identified as maps 2 and 3, respectively. Furthermore, maps 1 and 5 are rated as most similar by an intermediate number of users. In contrast, the interpretation of maps 4 and 6 is more controversial, because users supplied classification for both, similarity and dissimilarity. The given maps contain similar as well as dissimilar features and it depends on the subjectivity of the user on how to weight these. In this context, the subjectivity is desirable because it adds nuances to the overall pattern similarity score which is given in Table 3.

**Table 3:** Two examples that correspond with Fig 2. The most similar and dissimilar maps are classified by *n* users and the resulting pattern similarity score is calculated for each scenario.

Scenario	ET 2008; DOY 9			ET 2008; DOY 117		
	Similarity (n = 20)	Dissimilarity (n = 21)	Result	Similarity (n = 21)	Dissimilarity (n = 20)	Result
1	20	0	1.00	11	1	0.74
2	9	1	0.69	20	0	1.00
3	19	0	0.97	1	20	0.00
4	17	0	0.92	7	3	0.59
5	0	19	0.00	12	1	0.77
6	0	19	0.00	3	5	0.44

In the first example, the users unambiguously distinguish between similarity and dissimilarity whereas the second example is characterized with more ambiguity. This is reflected by the standard deviation of the six pattern similarity scores which is 0.43 and 0.32 for the first and second example, respectively. A higher standard deviation indicates a clear distinction between similar and dissimilar patterns whereas lower values suggest more nuances and diverging opinions among the users.

Obvious misclassifications are easy to identify; e.g. map 3 in example 2 (Table 3, DOY 117) received a single similarity classification versus 20 dissimilarity classifications. Potential misclassifications are not filtered in the analysis, because we expect these cases to be rare and to not significantly affect the final pattern similarity score.

### 3.3. Metric evaluation

**Fig 4:**
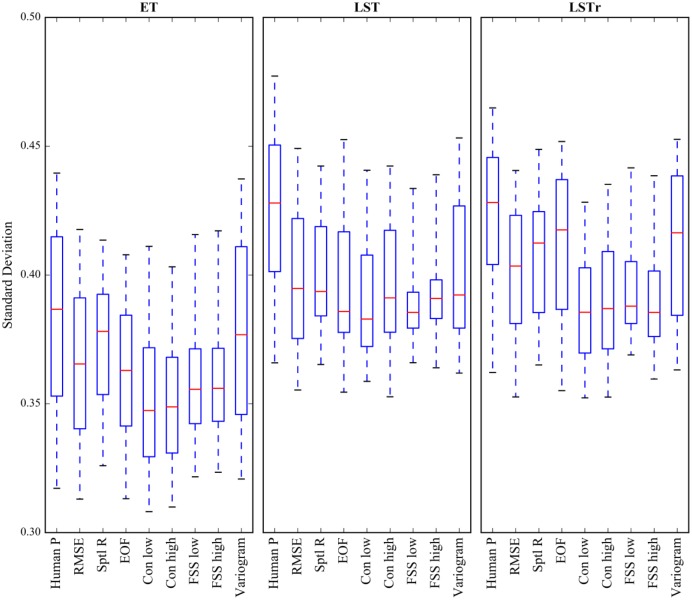
The ability of the human perception and metrics to differentiate between similar and dissimilar maps is investigated by means of the standard deviation of the six normalized pattern similarity scores at each of the 365 days per variable. The median is represented by the red line, the box indicates the 25^th^ and 75^th^ percentiles and the whiskers state the 5^th^ and 95^th^ percentiles.

The aim of the citizen science project is to benchmark a set of spatial performance metrics to evaluate their capabilities to mimic the human perception. Fig 4 provides first insights by showing the spatial similarity scores for simulated LST patterns based on the human perception and three spatial metrics for three scenarios: Scenario 1 that contains no spatial variability in precipitation, scenario 2 that reduces the spatial variability of the climate forcing and scenario 6 that spatially homogenizes the vegetation parametrization. A clear seasonality in the spatial similarity scores of the three scenarios can be attested by the human perception. Spatial variability in precipitation is regarded a minor driver of simulated LST patterns outside summer months, as indicated by high similarity scores. However, in case of rain events during the summer months, precipitation becomes the main driver of spatial variability of LST. Spatial variability in the vegetation parametrization is regarded sensitive during most of the year which is underlined by low pattern similarity scores. The spatial sensitivity of climate forcing with respect to simulated LST patterns is highly varying and a clear seasonal trend is less distinct. High pattern similarity scores and thus low sensitivities are generally evident in the summer months. On the contrary, climate forcing is often considered a major driver of spatial variability of LST during autumn and winter.

**Fig 5:**
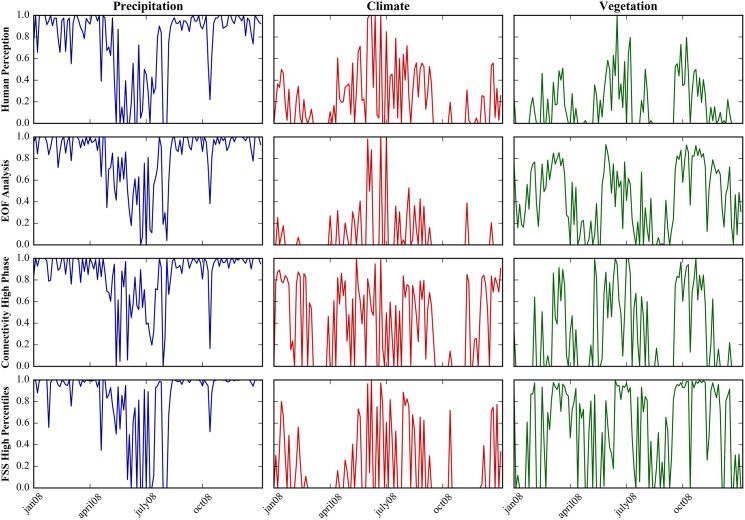
Evaluating spatial patterns of LST for 2008. The pattern similarity scores from the citizen science project are shown in the top panel. Panel two, three and four illustrate the pattern similarity scores for three selected performance metrics: EOF analysis, connectivity analysis of the high phase and FSS based on the high percentiles. Results are shown for three scenarios: Scenario 1 in blue (constant precipitation), scenario 2 in red (constant climate) and scenario 6 in green (constant vegetation).

Results obtained from EOF analysis, connectivity analysis and FSS are included in Fig 5 to visually inspect if the given metrics show similar characteristics with respect to the human perception. In order to be comparable, the pattern similarity scores from the performance metrics are normalized in the same manner as the human perception, i.e. by forcing the most similar scenario to 1 and the most dissimilar to 0. With respect to the human perception, the three shown metrics have comparable characteristics in terms of ranking the scenarios and their general seasonal trends. However, there are also distinct differences, such as the general higher similarity ranking of the vegetation scenario in spring and autumn by the metrics. Despite differences between the metrics and the human perception there are also ambiguous pattern similarity quantifications between the metrics.

Comparing spatial patterns is not a trivial task because it is required to take multiple dimensions of spatial information into account simultaneously. Typically, a spatial metric covers only a limited number of dimensions, such as the overall global structure or local variability. Therefore, metrics will naturally provide ambiguous results. The human perception is expected to be skilled at integrating several dimension of spatial information and thus can challenge a metric’s reliability and legitimacy. Table 4 states the coefficient of determination (R^2^) between the human perception based pattern similarity scores and the normalized metric scores. Most metrics explain more than 50% of the variance in the human perception based similarity scores. The R^2^ scores vary between the three variables, which indicates that the perturbations in the scenarios yield divergent alterations of the simulated spatial patterns to which both, the metrics and the human perception react differently. Overall, the RMSE, spatial correlation coefficient (spatial R), EOF analysis and variogram analysis explain the largest amounts of the variance in the human perception based similarity scores. It seems surprising that two simple cell-to-cell metrics are better at mimicking visual comparisons performed by humans than more advance metrics that provide more sophisticated pattern information. However, the way the scenarios are generated will ultimately only reduce the spatial variability and the applied perturbations are not expected to induce structural pattern mismatches. Therefore the patterns simulated by the scenarios cannot yield a complete re-arrangement of the baseline pattern, which promotes the cell-to-cell metrics. In consequence, these findings may be limited to the patterns incorporated in this study and for model evaluations against real spatial observations the spatial errors may be more complex and a simple RMSE may not be the most reliable metric. The lowest R^2^ scores are attested to the FSS metric, which may be caused by an inadequate choice of the critical scales that are subjectively defined for a number of thresholds. Despite the lower R^2^ scores in comparison to simpler metrics, the advanced metrics featured in this study stand out due to their flexibility and auxiliary information that potentially is beneficial in diagnosing spatial model deficiencies. For example, FSS and variograms are flexible in terms of scale and allow the modeller to assess the predictive capabilities of a model at a specific scale [51,52]. Furthermore the EOF analysis offers a combination of EOF maps and their associated loadings which provides valuable insights into the underlying processes that drive the spatio-temporal variability [16,53]. Lastly, connectivity is regarded a unique attributed of a spatial pattern [25] and can provide valuable diagnostics with respect to pattern structure and heterogeneity [23].

**Table 4:** The coefficient of determination (R^2^) between the human perception and the applied spatial performance metrics. The analysis is based on the daily spatial similarity scores for 365 days in the three year period (2008–2010) for all six scenarios.

R2	ET	LST	LST rise
	Human Perception		
RMSE	0.81	0.83	0.86
spatial R	0.78	0.73	0.78
EOF	0.76	0.70	0.84
Con Low	0.45	0.64	0.60
Con High	0.53	0.70	0.65
FSS Low	0.45	0.43	0.52
FSS High	0.53	0.53	0.40
Variogram	0.72	0.69	0.75

Aside from the correct ranking of scenarios with respect to their spatial similarity to the baseline model it is also of important that a metric is able to clearly distinguish between a good and a bad pattern agreement. As an example, this will state a clear advantage in a calibration of a hydrological model against spatial data where significant partial derivatives help the optimizer to efficiently find the optimum in the parameter space. The two examples in Fig 2 in section 3.2 show that the standard deviation between the six similarity scores can be consulted in order to quantify how ambiguous the spatial comparison is. Low values indicate nuances with several maps that show both, similar and dissimilar features whereas high values indicate a clear distinction between similar and dissimilar maps. This is addressed by Fig 5 that depicts box plots of the calculated standard deviations at 365 days for the human perception and the set of performance metrics for each variable. At first sight, the values for LST and LSTr are generally higher than for ET which indicates that distinguishing between similar and dissimilar spatial patterns is more straightforward in the former than in the latter. Assessing the box plots of all three variables underlines that the human perception provides the best breakdown of similar and dissimilar maps, which underlines that the effect of potential misclassifications can be considered to be very marginal. Overall, the connectivity analysis of the low phase has the lowest values suggesting that the similarity scores for the six maps are in close vicinity to each other. Taking all three variables into consideration, the variogram performs best in providing a clear distinction between similar and dissimilar maps. The variogram is a global measure that is not constrained by local agreement, and hence it can be suspected that the perturbations of the scenarios do not cause major displacements of the patterns. Therefore it can be assumed that the scenario’s effect on the spatial patterns is predominantly a disruption of the continuity in the data.

The list of applied metrics is clearly not exhaustive and the “perfect metric” is most likely not covered by this study. Presumably, a single metric will not be able to cover all relevant aspects of spatial information and instead a combination of metrics can be anticipated to be more informative. Alternatively, citizen science data can be utilized to train machine learning algorithms to analyse spatial patterns [54,55].

### 3.4. Spatial sensitivity analysis

**Fig 6:**
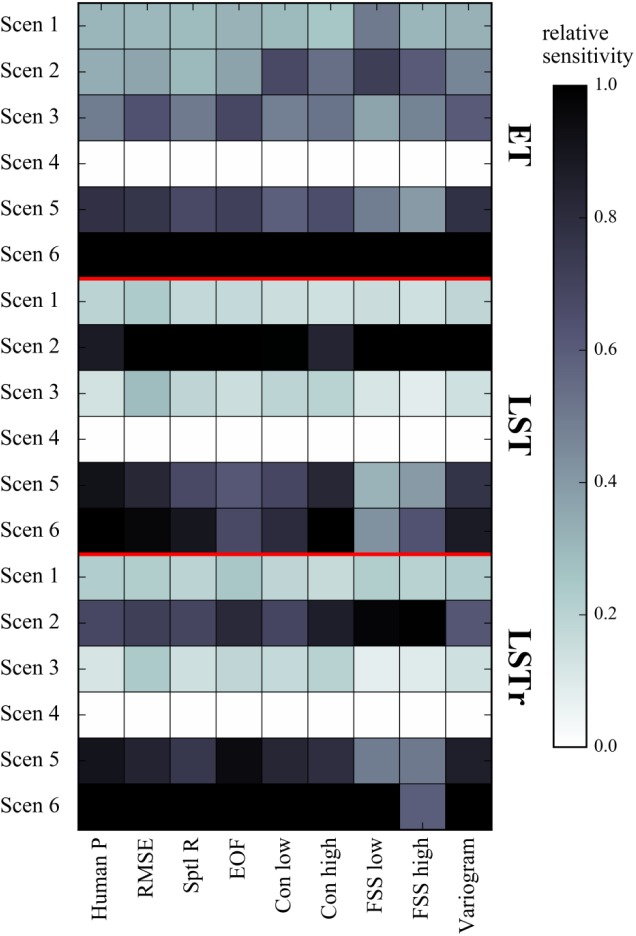
The relative sensitivity is given for the human perception and for the set of applied metrics. For each of the three variables, the sensitivity is linearly normalized relatively to the most sensitive scenario (1.0) and the least sensitive scenario (0.0).

In order to summarize the results of the scenario based spatial sensitivity analysis and to synthetize the overall comparison of the human perception and the applied metrics, Fig 6 shows the relative sensitivity for each scenario with respect to spatial patterns of ET, LST and LSTr. The relative sensitivity is calculated for both, the human perception and the set of applied performance metrics based on the average spatial similarity score over the entire three years. The scenario with the lowest average score is marked as most sensitive and the scenario with the highest average score is rated as the least sensitive scenario. The relative sensitivity for the remaining scenarios is linearly scaled between 0 and 1, where 1 defines the highest spatial sensitivity.

The set of applied metrics is overall in good agreement with the human perception. Hence differences between the scenarios and between the variables are more relevant at this stage to identify the most relevant drivers of spatial variability of the integrated catchment model of the Skjern catchment. The most prominent driver of spatial variability of all variables is the vegetation parametrization, where some metrics rate the 10 km smoothed dataset with an equal sensitivity compared to the constant dataset. This underlines that spatial information at 10 km scale does not necessarily provide better informative than having no spatial information at all. Spatial detail in climate forcing is most crucial for LST and LSTr which underlines the strong coupling to the atmospheric boundary layer. In general, the spatial sensitivity to groundwater coupling is more pronounced in ET which is rated similar throughout the metrics and the human perception. The least sensitive scenario for all variables is related to the spatial variability of the physical soil parameters in the unsaturated zone and the hydraulic conductivity distribution of the top geological layer. The geology of the Skjern catchment is described by soils that are predominantly characterized by various kinds of sandy soils with relatively similar soil physical properties. In other catchments that show a distinct heterogeneity in soil properties the findings may be different.

### 3.5. Citizen science

This study presents a novel approach to incorporate citizen science into hydrological science that goes beyond data collection in citizen observatories and instead employs the human perception to classify similarity of spatial patterns. In general, outsourcing visual tasks to humans is well studied in other fields of science [56–59]. Hydrology and other disciplines of environmental science heavily rely on the analysis of spatial data in form of observations or model outputs and the community can therefore benefit from the advances in citizen science featured by this study. In general, we do not propose to develop a citizen science project each time an evaluation of spatial patterns is required. Instead the community could benefit from a few benchmark datasets that contain a quantification of the human perception which can be utilized to test novel ways to assess spatial patterns.

This study makes use of existing internet based citizen science infrastructure, which allows scientists to easily implement citizen science as a tool in their work. In consequence, we do not claim to advance the field of citizen science; instead we aim at advancing the field of hydrology by introducing alternative ways to generate knowledge.

A clear and rigid definition of citizen science is not included in this study; however we do like to discuss a couple of distinctive features that are relevant for the conducted study. First, participants that voluntarily contribute to the collection or analysis of data can only be awarded as citizen scientists if they themselves are not subject of the study. This delineates citizen science from other, more traditional areas of science where volunteers participate in social science surveys or medical trials [60]. Moreover it is important to reflect on nuances between crowdsourcing and citizen science, because not all crowdsourcing efforts necessarily fall under the category of citizen science. The openness and the degree to which the scientist engage with the users mark the main criteria which has to be complied in crowdsourcing studies in order to be referred to as citizen science [2]. According to this, multiple studies where so called “clickworkers” perform very simple visual task of data analysis can be labelled as citizen science efforts [4,6,61]. Despite disclosure and active engagement, the rationale behind this classification is supported twofold, first due to the objective to educate the public about scientific issues and secondly due to the premise that the volunteers have some sort of superior capacities over computer based solutions. On the contrary, Franzoni and Sauermann [2] brought forward several examples of crowdsourcing efforts that do not aim at disclosure and which thus cannot be classified as citizen science projects. Following the abovementioned classification *Pattern Perception* can be categorized as citizen science.

## 4. Conclusion

The recently gain popularity and the technological advances of citizen science inspire to reformulate traditional scientific practice by complementing it with new sources of data and model evaluation methods. Well established citizen science infrastructures, such as Zooniverse, allow scientists to efficiently build projects that can potentially attract a large number of volunteers. We have initiated *Pattern Perception*, a citizen science project that employs visual perception skills of the Zooniverse users to evaluate similarity and dissimilarity of spatial patterns simulated by an integrated hydrological model for a catchment in Denmark. We can summarize three major conclusions from our work:

- The self-proclaimed goal of at least 20 classifications for each of the 2190 subjects was reach in just 64 days with contributions from 2,898 users. The distribution of classifications per user was found to be less skewed than it has been documented for other Zooniverse projects. *Pattern Perception* is based on very well-defined tasks that can be completed swiftly by the user, which may have provoked this favourable reduction in skewness.
- The ability of a set of spatial performance metrics to mimic the human perception is addressed in this study and results indicate that more complex metrics do not necessarily perform better at this task than simple cell-to-cell metrics. However, more advance metrics provide flexibility and auxiliary information which may be favourable over simpler metrics. Moreover, the perturbation design favours cell-to-cell metrics, because no major misplacements in the patterns are induced by the scenarios. Generally, the metrics vary in their competence to unambiguously identify similar and dissimilar scenarios, i.e. the variogram analysis is most conclusive and the connectivity analysis of the low phase is most vaguely.
- The scenario based spatial sensitivity analysis provides insights into the driving mechanism behind spatial patterns at the land-atmosphere interface. The human visual perception in combination with the set of metrics allow drawing comparable conclusions with respect to the most sensitive scenarios. Spatial patterns of all three variables are strongly influenced by spatial detail in the vegetation parametrization. LST and LSTr are explicitly linked to the atmospheric conditions via spatial variability in the climate forcing whereas ET is rather driven by spatial patterns in the precipitation input and the groundwater coupling.

Lastly, we hope to encourage others to utilize this unique dataset that contains pattern similarity scores derived from the human perception for over 1,000 sextets of spatial patterns with respective reference patterns. This dataset can be appointed as a benchmark and hence support the community in deciding which spatial performance metric to trust. The data is made available via GitHub (https://github.com/JulKoch/SEEM).
